# Novel clipping procedure for preventing post‐operative inguinal hernia in robot‐assisted radical prostatectomy

**DOI:** 10.1111/iju.15544

**Published:** 2024-08-09

**Authors:** Yuji Hakozaki, Yuta Yamada, Tetsuya Fujimura, Naoki Kimura, Kenichi Sasaki, Kazuki Maki, Kazuma Sugimoto, Taro Izumi, Jun Kaneko, Fumihiko Urabe, Mayuko Tokunaga, Yoichi Fujii, Jun Kamei, Taketo Kawai, Satoru Taguchi, Yoshiyuki Akiyama, Daisuke Yamada, Haruki Kume

**Affiliations:** ^1^ Department of Urology The University of Tokyo Graduate School of Medicine Tokyo Japan; ^2^ Department of Urology Jichi Medical University Tochigi Japan; ^3^ Department of Urology National Center for Global Health and Medicine Tokyo Japan; ^4^ Department of Urology Kikkoman General Hospital Chiba Japan; ^5^ Department of Urology Yashio Central General Hospital Saitama Japan; ^6^ Department of Urology Tokyo Metropolitan Tama Medical Center Tokyo Japan; ^7^ Department of Urology Jikei University School of Medicine Tokyo Japan; ^8^ Department of Urology Teikyo University School of Medicine Tokyo Japan

**Keywords:** inguinal hernia, prophylactic procedure, prostate cancer, robot‐assisted radical prostatectomy, surgical experience

## Abstract

**Objectives:**

Inguinal hernia (IH) is a common postoperative complication after robot‐assisted radical prostatectomy (RARP). We developed a novel clipping technique for the prevention of IH developing after RARP.

**Methods:**

This cohort included 759 consecutive patients who underwent RARP for prostate cancer at the University of Tokyo Hospital between January 2011 and December 2018. We reviewed clinical parameters and identified the risk factors of postoperative IH. The prophylactic preventive procedure of IH development was performed by clipping the peritoneum and underlying tissue around the internal inguinal ring using Hem‐o‐Lok clip to prevent the prolapse of the intestine through the internal inguinal ring.

**Results:**

In total, 236 patients received the clipping procedure. The median follow‐up time was 50 months. The incidence rate of IH was 10.8% (78/720). The median time to the diagnosis of IH was 10 months. Univariate analysis revealed that patients with higher age (age ≥ 63), low BMI (BMI < 25 kg/m^2^), and lower number of surgical experiences (Surgical experience < 40) showed a significantly higher odds ratio of developing IH. Multivariate analysis showed that “BMI < 25 kg/m^2^” and “Surgical experience < 40” were independent predictive factors of IH. Among the patients with a high risk of IH due to receiving surgery from inexperienced surgeons, there was a statistically significant preventive effect for the patients with “BMI ≥ 25 kg/m^2^” by the novel clipping procedure.

**Conclusions:**

The novel clipping procedure reduced the risk of post‐operative IH in obese patients when the RARP was performed by inexperienced surgeons.

**Abbreviations & Acronyms**
IH: inguinal hernia
IPSS: International Prostate Symptom Score
ORP: open radical prostatectomy
PPV: patent processus vaginalis
RARP: robot‐assisted radical prostatectomy
RRP: radical retropubic prostatectomy

## INTRODUCTION

Inguinal hernia (IH) is a prolapse of the intestine bulging out the internal inguinal ring. In 1996, Regan et al. reported that 12% of patients developed IH within 6 months after open radical prostatectomy (ORP), which is a higher incidence compared to the incidence rate in the general male population (5%).^1^ Subsequently, several reports have shown that ORP is associated with a risk of IH,^2^, ^3^, ^4^ which is consistent with the fact that laparoscopic surgery with minimum incision improved the rate of postsurgical IH compared to conventional radical retropubic prostatectomy (RRP).^5^ It is interesting to see that laparoscopy‐assisted radical prostatectomy with minimum incision reduced the rate of IH incidence compared to conventional open radical prostatectomy. Given this, reducing the invasiveness of the surgery may have affected the incidence of postoperative incidence of IH. Indeed, in a less invasive approach such as robot‐assisted radical prostatectomy (RARP).

Several reports showed that RARP decreased the risk of IH compared to ORP.^6^, ^7^, ^8^ However, there remains a risk of developing IH for the patients even in cases with RARP procedure,^9^ and IH develops in approximately 3% to 14% of patients after this procedure.^6^, ^8^, ^9^

We developed a novel procedure for the prevention of IH. We describe the process of the procedure and evaluate whether this technique can reduce the incidence of IH.

## METHODS

### Study design

This cohort included 759 consecutive patients who underwent RARP for prostate cancer at the University of Tokyo Hospital between January 2011 and December 2018. Surgeries were conducted by 23 different urologists over 7 years. This study was performed in accordance with the Declaration of Helsinki and approved by the “Ethics Committee of the Tokyo University Hospital” (approval number #2020039NI), and all the patients in the present cohort provided written informed consent before RARP.

### Patient selection

To assess the benefit of the prevention procedure for each patient, we excluded two patients who received the unilateral procedure. In the cohort, 37 patients had a history of unilateral or bilateral IH. Those patients were excluded to evaluate the risk of IH for treatment naïve patients. In total, 720 patients were included in the analysis (Figure S1).

### IH preventive procedure

**FIGURE 1:**
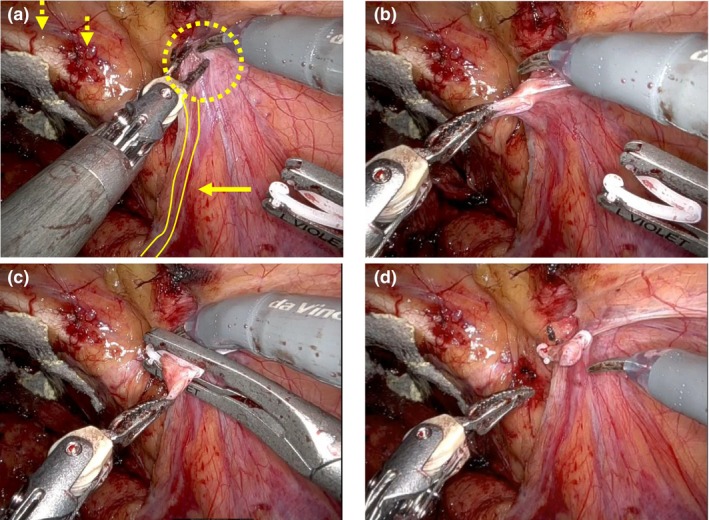
Representative images of the novel clipping procedure during robot‐assisted radical prostatectomy (a) The peritoneum and underlying tissue were grasped around the right internal inguinal ring (dotted circle), Vas deferens (arrow), and pubis (dotted arrow). (b–d) The grasped peritoneum and underlying tissue were clipped using a large‐size hem‐o‐Lok clip.

We developed a novel procedure for preventing IH after RARP. This prevention procedure was performed after the incision of the peritoneum medial to the internal inguinal ring and alongside the vas deferens (Figure 1a; Video S1). The peritoneum in the center of the internal inguinal ring was grasped (Figure 1b). The grasped peritoneum and underlying tissue were clipped to prevent the development of prolapse of the intestine through the internal inguinal ring using a large‐size Hem‐o‐Lok clip (Teleflex, PA, USA) (Figure 1c,d). The prevention procedure was performed for the patients who showed consent to the prophylactic intervention. No other prophylactic interventions were performed such as cutting the vas deferens at the orifice of the internal inguinal ring.

### Data retrieval

The clinical data was collected from the medical record. Reported risk factors of IH were collected, including BMI,^10^ age,^7^ International Prostate Symptom Score (IPSS) score,^11^ and number of surgical experience.^12^ In addition to these risk factors, preoperative PSA was collected. Data related to the surgery was collected, including pathological T stage, resected prostate weight, operative time, console time, number of surgical experiences of RARP procedures, presence of nerve sparing, and blood loss. Postoperative data was collected, including the date of IH diagnosis and the type of IH. Inguinal hernia‐free survival is defined as the time from the date of the surgery until the diagnosis of IH. Adverse events involving Hem‐o‐Lock clipping were collected from medical cords.

### Statistical analysis

Clinical parameters were shown by the frequency and percentage of categorical variables or the median value attached to the IQR for continuous variables. Patients were divided into “prevention group” and “non‐prevention group” according to the presence of a preventive procedure. Continuous and categorical variables between the two groups were compared using the Mann–Whitney U test and Fisher's exact test, respectively. Univariate analysis was performed using the Cox proportional hazards regression model to identify risk factors predicting an inguinal hernia‐free survival. Regarding continuous variables, the cutoff values were determined by the Cutoff finder software,^13^ which determined the optimal cutoff with the most significant split in the log‐rank test for the incidence of the IH. All *p* values with <0.05 were considered statistically significant. Statistically significant prognostic factors in the univariate analysis were included in the multivariate analysis. The Kaplan–Meier curves of an inguinal hernia‐free survival were constructed. All statistical analysis was performed using R Version 4.0.3 (Comprehensive R Archive Network).

## RESULTS

### Patient characteristics

The patient characteristics are shown in Table 1 according to the presence of the preventive procedure. In total, 236 patients received the clipping procedure for prevention of IH. Patients who received the clipping procedure (prevention group) showed greater prostate weight, higher surgical experiences, less blood loss, and shorter follow‐up period. Among the reported risk factors of IH, the prevention group showed more surgical experiences.

**Table 1:** Characteristics of the patients receiving robot‐assisted radical prostatectomy.

Variables	Prevention group (*n* = 236)	Non‐prevention group (*n* = 484)	*p*‐value
Age, years	68 (64–73)	67 (64–71)	0.095
BMI, kg/m^2^	24 (22–26)	24 (22–26)	0.501
PSA, ng/mL	7.3 (5.8–11)	7.7 (5.5–11)	0.871
Prostate weight, g	42 (37–52)	39 (28–50)	<0.001*
pT stage, *n*			
pT2a‐T2c	170 (72.0)	326 (67.4)	0.230
≥pT3	66 (28.0)	158 (32.6)	
IPSS score	6 (3–12)	7 (3–11)	0.479
Straining	103 (44.2)	182 (38.2)	0.142
Operative time, min	216 (176–273)	230 (188–264)	0.322
Console time, min	160 (125–209)	171 (134–208)	0.121
Surgical experience, *n*	28 (13–72)	25 (10–46)	0.007*
Blood loss, mL	250 (100–400)	300 (130–570)	0.001*
Nerve sparing	72 (30.5)	136 (28.1)	0.540
Duration of follow‐up time, months	48 (35–57)	53 (36–66)	0.001*

*Note*: Median value (IQR) or *N* (%). Associations between continuous variables were compared by the Mann–Whitney *U* test and the difference of categorical values was compared by Fisher's exact test.

Abbreviations: BMI, body mass index; PSA, prostate‐specific antigen; IQR, interquartile range.

* Statistically significant with a *p* < 0.05.

### Postoperative inguinal hernia

**FIGURE 2:**
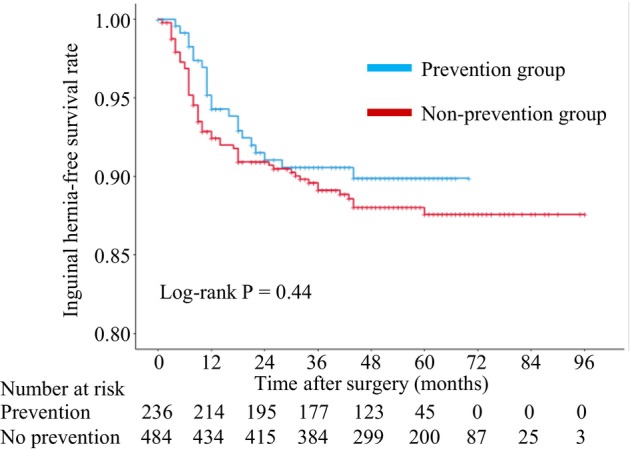
Kaplan–Meier curves predicting inguinal hernia‐free survival (prevention group vs. non‐prevention group).

The median follow‐up time from the date of surgery was 50 months. The incidence rate of IH was 10.8% (78/720). The median time to the diagnosis of the IH from the date of surgery was 10 months. Among 78 patients with postoperative IH, 70 (89%) patients were diagnosed within 3 years of RARP. Among these patients, 9 (12%) patients were diagnosed with bilateral IH (Table S1). With regards to the laterality of the IH, 46 (59%) and 22 (28%) patients showed right and left IH, respectively. The incidence rate of IH was 9.3% (22/236) in the prevention group and 11.6% (56/484) in the non‐prevention group, respectively. There were no adverse events relating to clipping procedures such as clip migration or postoperative groin pain. Kaplan–Meier estimates of developing an IH at 36 months were 9.4% in the prevention group and 10.9% in the non‐prevention group, respectively (Figure 2). There is no statistically significant difference according to the presence of the preventive procedure (*p* = 0.44).

### Risk stratification of IH

Univariate analysis revealed that patients with low BMI (BMI < 25 kg/m^2^), and a lower number of surgical experiences (Surgical experience <40) showed significantly higher odds ratio of the IH development (Table 2). Multivariate analysis showed that “BMI < 25 kg/m^2^” and “Surgical experience < 40” were independent predictive factors of IH.

**TABLE 2:** Univariate and multivariate analysis of prognostic factors for developing postoperative inguinal hernia.

Factors	Univariate analysis		Multivariate analysis	
	Hazard ratio (95%CI)	*p*‐value	Hazard ratio (95%CI)	*p*‐value
Clipping procedure (yes vs. no)	0.82 (0.50–1.35)	0.436		
Age (≥63 vs. ≤63 years)	1.83 (0.94–3.56)	0.074		
PSA (>16 vs. ≤16 ng/mL)	0.46 (0.17–1.27)	0.134		
BMI (<25 vs. ≥25 kg/m^2^)	3.13 (1.78–5.50)	<0.001*	3.21 (1.83–5.64)	<0.001*
Prostate weight (>50 vs. ≤50 g)	1.56 (0.97–2.53)	0.068		
IPSS (>3 vs. ≤3)	1.47 (0.78–2.78)	0.235		
Straining (positive vs. negative)	1.01 (0.64–1.59)	0.983		
Operative time (>140 vs. ≤140 min)	0.78 (0.31–1.91)	0.577		
Console time (>200 vs. ≤200 min)	1.46 (0.93–2.31)	0.104		
Surgical experience (<40 vs. ≥40)	2.03 (1.17–3.51)	0.012*	2.12 (1.22–3.67)	0.008*
Blood loss (>500 vs. ≤500 mL)	0.67 (0.37–1.21)	0.183		
Nerve preservation (yes vs. no)	1.36 (0.78–2.37)	0.277		

Abbreviations: BMI, body mass index; CI, confidence interval; PSA, prostate‐specific antigen.

* Statistically significant with a *p* < 0.05.

### Effect of prevention for the patients stratified by the risk factors of IH

**FIGURE 3:**
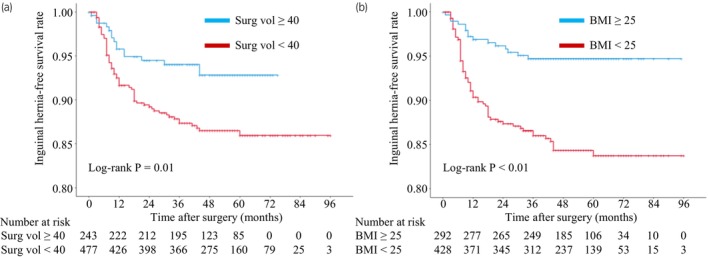
Kaplan–Meier curves predicting inguinal hernia‐free survival based on the surgical volume of RARP and body mass index.

The patients were divided according to the IH risk using the identified predictive factors followed by the assessment of the preventive effect of the clipping procedure. The percentages of patients developing IH receiving surgeries from surgeons with “surgical experience < 40” and “surgical experience ≥ 40” were 13.0% (62/477) and 6.6% (16/243), respectively (Figure 3a). The incidence rates of IH for the patients with “BMI < 25 kg/m^2^” and “BMI ≥25 kg/m^2^” were 16.4% (70/428) and 2.7% (8/292), respectively (Figure 3b).

**FIGURE 4:**
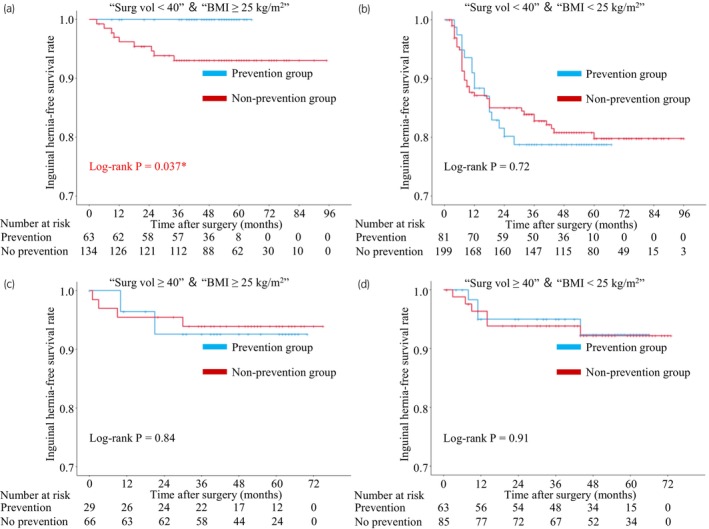
Kaplan–Meier curves predicting inguinal hernia‐free survival based on the postoperative inguinal hernia risk factors. (a) “Surg vol < 40” and “BMI ≥ 25 kg/m^2^” (b) “Surg vol < 40” and “BMI < 25 kg/m^2^” (c) “Surg vol ≥ 40” and “BMI ≥25 kg/m^2^” (d) “Surg vol ≥ 40” and “BMI < 25 kg/m^2^.” BMI, body mass index; Surg vol, surgeon volume.

Among patients with high IH risk with “surgical experience < 40”, there was a statistically significant preventive effect in patients with “BMI ≥ 25 kg/m^2^” (Figure 4a) by performing this clip‐procedure. On the other hand, there was no preventive effect in patients with “BMI < 25 kg/m^2^” (Figure 4b). There was no significant difference in the risk of IH development between patients with BMI ≥25 kg/m^2^ and BMI < 25 kg/m^2^ in a subpopulation of patients undergoing surgery performed by well‐experienced surgeons (Figure 4c,d).

## DISCUSSION

We described the novel clipping technique for preventing IH after RARP and evaluated whether this technique can reduce the incidence of IH. Low BMI and less surgeon‐experience of RARP were identified as risk factors for the incidence of IH. By dividing the risk according to these identified factors, this clipping technique provided the benefit of preventing IH development in cases associated with high BMI and less surgeon experience.

Several risk factors of IH development after RARP, include BMI,^14^, ^15^ psoas muscle volume,^16^ patent processus vaginalis,^11^, ^17^ surgical experience,^12^ and IPSS score.^11^ Low BMI is a well‐established IH risk factor for patients undergoing RARP.^14^, ^15^ The psoas muscle volume positively correlated with the BMI value, which was reported as a risk factor for IH.^16^ Patent processus vaginalis is also associated with a high risk of developing IH after RARP. Majima et al. reported that patients with patent processus vaginalis (PPV) showed a high rate of IH development compared to patients with negative PPV (37% vs. 4.8%).^11^

We identified low surgical experience as an IH risk factor after RARP, and the percentage of patients developing IH receiving surgeries from not‐well‐experienced surgeons was approximately two times (13.0% vs. 6.6%) compared to well‐experienced surgeons. We have previously reported that low surgical experience is an IH risk factor,^12^ and this was also the case when the number of participants was increased by double. Not well‐experienced surgeons may excessively spread unnecessary working space while exposing the Retzius space, which leads to tissue damage around the internal inguinal orifice.^12^ A report by Iwamoto et al. has shown a reduced incidence rate of IH development when retroperitoneal space lateral to the medial umbilical ligament is preserved during peritoneal incision.^17^

We identified low “BMI < 25 kg/m^2^” as an independent predictor of IH, which was in line with the previous report.^14^ The incidence rate of developing IH was only 3.4% in patients with “BMI ≥ 25” whereas it was 14.4% in patients with “BMI < 25.” Zendejas et al. described the possible reason as excess intra‐abdominal or pre‐peritoneal fat could provide a barrier effect by acting as a “plug” to prevent herniation of abdominal contents.^18^ With regards to our novel clipping technique, the clip and the connective tissue around it with adhesion may provide an extra‐supportive barrier acting as a “plug” on top of the internal inguinal ring.

Our novel clipping technique provided IH‐preventative benefits in patients with high BMI when RARP was performed by low‐experienced surgeons. Several possible explanations have been postulated for the preventative effect for high BMI patients: (1) the strength of tension on the clipped supporting tissues surrounding the internal inguinal ring may be stronger for the high BMI patients.^19^ (2) adhesion around the internal inguinal ring prevents the prolapse of the intestine as the higher risk of intra‐abdominal adhesion for the patients. Notably, high BMI was associated with a high incidence rate of adhesion reported in women undergoing repeat cesarean sections.^20^ Additionally, Iwamoto et al. reported that incising the peritoneum close to the medial edge of the internal inguinal ring decreased the incidence rate of IH.^17^ They assessed that the inflammatory adhesions contributed to the prevention of IH.^17^ On top of this, a high degree of adhesion may occur by using the Hem‐o‐loc clip, as this was demonstrated in an animal model by Bajric et al.^21^

Several prophylactic methods to prevent IH after RARP were reported and performing these measures achieved a significantly decreased rate of IH development.^22^, ^23^, ^24^ The common process of these procedures was dissecting the peritoneum around the internal ring, whereas the following procedures were different: cutting the vas deferens,^22^ suturing the dissected peritoneum,^23^ and plugging a hemostatic agent into the inguinal canal.^24^

The ratio of IH laterality and the development of indirect IH were similar to the previous study.^25^ Agarwal PK et al. reported that the most common type of inguinal hernia was indirect (60%) compared to direct (30%) or bilateral (10%), and the most common side of hernia was right (63%) compared to the left (33%) or bilateral (4%).^25^ It is interesting that these results were similar to our data.

There were several limitations according to the study. First, this is a retrospective cohort and the prevention group is not randomly assigned. We stratified IH risk and analyzed the risk of IH according to the group. However, there might be a selection bias to perform clipping procedures. Second, postoperative IH type is unknown in approximately 30% of all cases, although it can be easily expected that IH developing in post‐RARP is mostly indirect hernias. Notably, our prophylactic procedure was mainly focused on preventing the development of indirect IH.

Our novel clipping procedure has several attractive points compared to the previous prophylactic methods. First, it takes minimal time with less than 30 s. Second, this procedure does not require dissection beyond the internal inguinal ring which may provide an advantage during hernioplasty that may be performed when IH develops in the future. Third, it is cost‐effective since this procedure only uses one or two clips. However, the method seems to have a weak preventive effect in patients with low BMI patients, given that this spectrum of patients harbors too great a risk (Hazard ratio: 3.21) of developing IH and thus cannot receive sufficient benefits from this procedure.

In this study, the increased risk of IH due to a lack of surgical experience was reduced with the developed novel clipping procedure. However, patients with low BMI are still at higher risk of developing IH, and the clipping procedure in the present study was not sufficient for these patients.

## AUTHOR CONTRIBUTIONS

**Yuji Hakozaki:** Conceptualization; data curation; formal analysis; writing – original draft. **Yuta Yamada:** Conceptualization; data curation; methodology; writing – original draft; writing – review and editing. **Tetsuya Fujimura:** Conceptualization; data curation; writing – review and editing. **Naoki Kimura:** Writing – review and editing. **Kenichi Sasaki:** Conceptualization; data curation. **Kazuki Maki:** Writing – review and editing. **Kazuma Sugimoto:** Writing – review and editing. **Taro Izumi:** Methodology. **Jun Kaneko:** Writing – review and editing. **Fumihiko Urabe:** Writing – review and editing. **Mayuko Tokunaga:** Methodology. **Yoichi Fujii:** Writing – review and editing. **Jun Kamei:** Writing – review and editing. **Taketo Kawai:** Writing – review and editing. **Satoru Taguchi:** Writing – review and editing. **Yoshiyuki Akiyama:** Writing – review and editing. **Daisuke Yamada:** Writing – review and editing. **Haruki Kume:** Writing – review and editing.

## CONFLICT OF INTEREST STATEMENT

Tetsuya Fujimura and Haruki Kume are the Editorial Board members of International Journal of Urology and the co‐authors of this article. To minimize bias, they were excluded from all editorial decision‐making related to the acceptance of this article for publication.

## APPROVAL OF THE RESEARCH PROTOCOL BY AN INSTITUTIONAL REVIEWER BOARD

Obtained; approval no. 2020039NI.

## INFORMED CONSENT

All the patients in the present cohort provided written informed consent.

## REGISTRY AND THE REGISTRATION NO. OF THE STUDY/TRIAL

N/A.

## ANIMAL STUDIES

N/A.

## Supporting information


Data S1.
