# Supplementary material for: Novel clipping procedure for preventing post‐operative inguinal hernia in robot‐assisted radical prostatectomy
Source: Int J Urol. 2024 Aug 9;31(11):1241–7. doi: 10.1111/iju.15544 (PMC11867017; doi:10.1111/iju.15544)
Supplement: Supplementary file 1 — Data S1. [file IJU-31-1241-s001.zip › iju15544-sup-0001-FigureS1.pptx]

## Slide 1
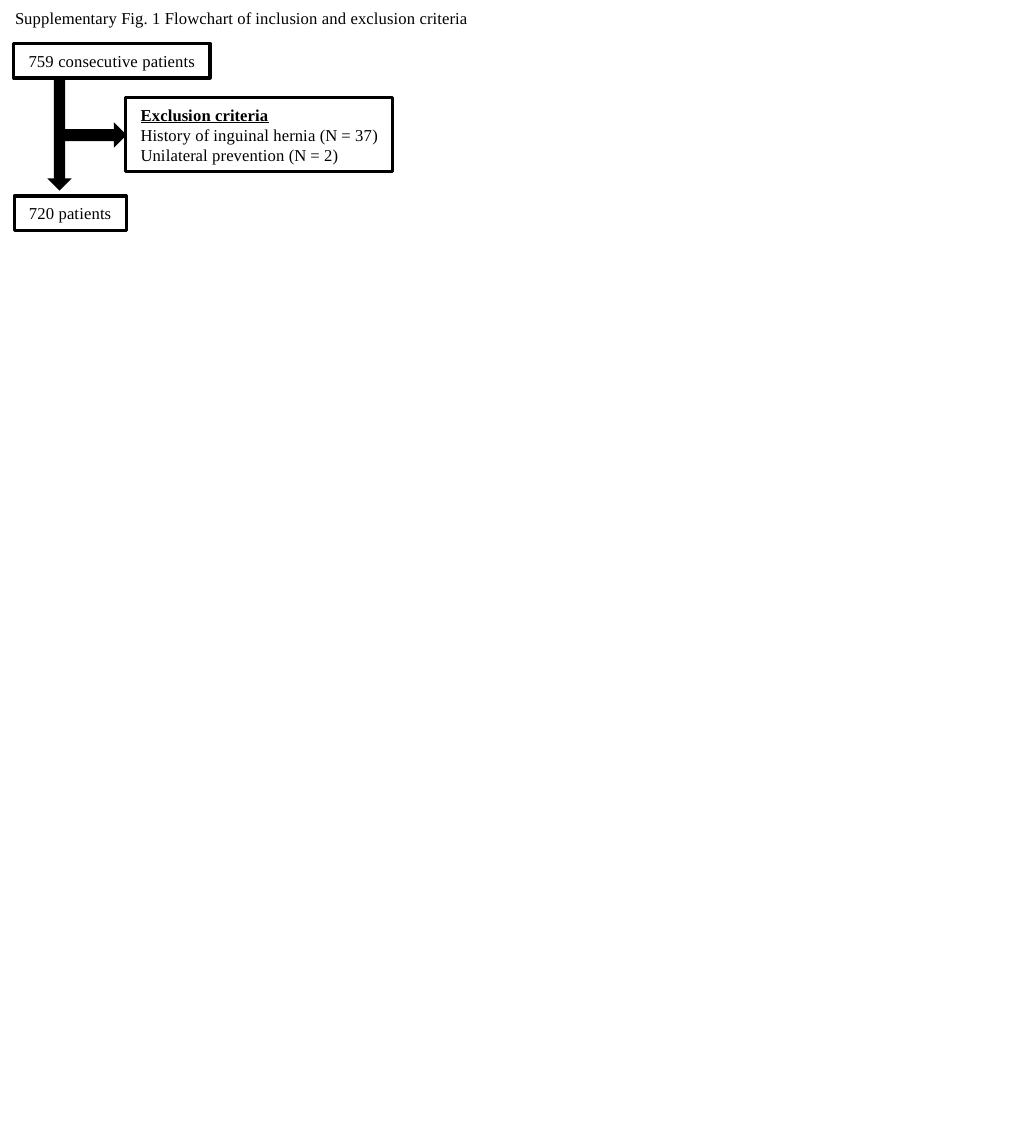

Supplementary Fig. 1 Flowchart of inclusion and exclusion criteria
759 consecutive patients
Exclusion criteria
History of inguinal hernia (N = 37)
Unilateral prevention (N = 2)
720 patients
