# Supplementary material for: Novel clipping procedure for preventing post‐operative inguinal hernia in robot‐assisted radical prostatectomy
Source: Int J Urol. 2024 Aug 9;31(11):1241–7. doi: 10.1111/iju.15544 (PMC11867017; doi:10.1111/iju.15544)
Supplement: Supplementary file 1 — Data S1. [file IJU-31-1241-s001.zip › iju15544-sup-0002-TableS1.docx]

**Supplementary table 1** Features of postoperative inguinal hernia

| Variables | N (%) |
| --- | --- |
| No. of postoperative hernia | 78 (100) |
| Laterality Right  　　Left  Bilateral  Unknown | 46 (59.0)  22 (28.2)  9 (11.5)  1 (1.3) |
| Type Indirect  Direct  Unknown | 48 (61.5)  3 (3.8)  27 (34.6) |
